# Retrospective analysis of wildfire smoke exposure and birth weight outcomes in the San Francisco Bay Area of California

**DOI:** 10.1088/2752-5309/acd5f5

**Published:** 2023-06-13

**Authors:** Anna Claire G Fernández, Emilia Basilio, Tarik Benmarhnia, Jacquelyn Roger, Stephanie L Gaw, Joshua F Robinson, Amy M Padula

**Affiliations:** 1 School of Public Health, University of California, Berkeley; 2 School of Medicine, University of California, San Francisco; 3 Department of Obstetrics, Gynecology and Reproductive Sciences, University of California, San Francisco; 4 Scripps Institution of Oceanography, University of California, San Diego

**Keywords:** wildfires, PM_2.5_, pregnancy, birth weight, fetal growth

## Abstract

Despite the occurrence of wildfires quadrupling over the past four decades, the health effects associated with wildfire smoke exposures during pregnancy remains unknown. Particulate matter less than 2.5 *μ*ms (PM_2.5_) is among the major pollutants emitted in wildfire smoke. Previous studies found PM_2.5_ associated with lower birthweight, however, the relationship between wildfire-specific PM_2.5_ and birthweight is uncertain. Our study of 7923 singleton births in San Francisco between January 1, 2017 and March 12, 2020 examines associations between wildfire smoke exposure during pregnancy and birthweight. We linked daily estimates of wildfire-specific PM_2.5_ to maternal residence at the ZIP code level. We used linear and log-binomial regression to examine the relationship between wildfire smoke exposure by trimester and birthweight and adjusted for gestational age, maternal age, race/ethnicity, and educational attainment. We stratified by infant sex to examine potential effect modification. Exposure to wildfire-specific PM_2.5_ during the second trimester of pregnancy was positively associated with increased risk of large for gestational age (*OR* = 1.13; 95% CI: 1.03, 1.24), as was the number of days of wildfire-specific PM_2.5_ above 5 *μ*g m^−3^ in the second trimester (*OR* = 1.03; 95% CI: 1.01, 1.06). We found consistent results with wildfire smoke exposure in the second trimester and increased continuous birthweight-for-gestational age *z*-score. Differences by infant sex were not consistent. Counter to our hypothesis, results suggest that wildfire smoke exposures are associated with increased risk for higher birthweight. We observed strongest associations during the second trimester. These investigations should be expanded to other populations exposed to wildfire smoke and aim to identify vulnerable communities. Additional research is needed to clarify the biological mechanisms in this relationship between wildfire smoke exposure and adverse birth outcomes.

## 1. Introduction

Both wildfire size and intensity have quadrupled in the last four decades nationwide (National Interagency Fire Center 2021). Moreover, the combined effect of the increasing land area burned by wildfires and human development into previously uninhabited forest areas increase the risk of morbidity and mortality associated with wildfires. From 1990–2010, the number of homes that exist on the wildfire-urban interface, the area between wilderness and developed land, grew 41% (Radeloff *et al* 2018). With this increased proximity to wildfires and wildfire smoke comes an increasing threat posed to air quality, and by proxy, public health. In recent years, wildfires have accounted for 25% of particulate matter found in air pollution measuring less than 2.5 *μ*ms (PM_2.5_) nationwide and have accounted for nearly 50% of PM_2.5_ in the Western United States (Burke *et al* 2021). As wildfires become more pervasive, it is imperative to understand how they affect pregnancy, fetal development, and birth outcomes.

Developing fetuses depend on maternal blood for access to maternal oxygen, which is essential to their development. PM_2.5_ has been shown capable of crossing the placental barrier (Wick *et al* 2010, Bové *et al* 2019) which serves a crucial role in selectively filtering maternal blood for oxygen delivery to the fetus. Recent studies demonstrate how maternal PM_2.5_ exposure not only inhibits the proliferative capability of placenta cells, but also alters nutrient transporter expression in the placenta and thus disrupts maternal-fetal nutrient transportation (Zhu *et al* 2021). Additionally, PM_2.5_ exposure has been associated with modulated expression of genes crucial to inflammation, lipid transport, and cell-to-cell communication. These metabolic effects are also thought to be sex-dependent, with males at higher risk of metabolic alteration in the context of increased PM_2.5_ exposure (Kaur *et al* ). Male fetuses are also more vulnerable to fetal loss and adverse birth outcomes, especially in the context of maternal stress (Kaitz *et al* 2015) and increased exposure to PM_2.5_ (Zhang *et al* 2019). This proposed process may provide a causative mechanism by which to explain the adverse effects on birth weight associated with PM_2.5_ exposure.

The effect of PM_2.5_ on birth weight is crucial because newborn weight serves as a predictor of adverse health outcomes later in life. A recent review demonstrated an increased risk of future risk of cardiovascular disease, cancer, respiratory illness and chronic metabolic conditions among low birthweight infants (Belbasis *et al* 2016). Additionally, babies born large for gestational age (LGA)—above the 90th percentile for weight given gestational age—are at risk of trauma secondary to complicated vaginal deliveries (e.g. shoulder dystocia) and conditions like hypoglycemia, independent of whether gestational diabetes was present during pregnancy (O’Donnell and Behie 2015).

Wildfires are thought to influence birth weight in complex ways, and researchers have yet to reach consensus on the exact impact of PM_2.5_ exposure on neonatal birth weight. It remains unclear whether wildfire smoke exposure impacts birth weight and in which direction. For example, there is evidence from a review to indicate that PM_2.5_ exposure is associated with reduced birth weight (Amjad *et al* 2021). This effect seems temporally-specific and impacted by the trimester in which smoke exposure occurred: the majority of studies reviewed on this topic reported a reduction in newborn birth weight following wildfire exposure in the second and third trimesters. Certainty in these results were low, however, due to limited ability to capture wildfire-specific sources of PM_2.5_ at the exclusion of other pollution sources and difficulty estimating distances from wildfire areas throughout pregnancy (Amjad *et al* 2021).

Conversely, an Australian study found that increased exposure to PM_2.5_ in wildfire smoke correlated with increased birth weight when compared to pregnancies unexposed to wildfires. This result was sex-specific: only males born to exposed mothers were born large for their gestational age, while the trend was not observed for females in the same cohort (O’Donnell and Behie 2015). This may be due to the sexually dimorphic nature of fetal growth in the response to physiological stress (Clifton 2010). While female fetuses tend to conserve growth in response to maternal stress, male fetuses often demonstrate increased metabolic activity and prioritization of growth in less optimal maternal environments (Kaitz *et al* 2015), often leading to more complications and adverse outcomes like intrauterine growth restriction, preterm delivery, or death (Di Renzo *et al* 2007).

To understand how environmental exposures lead to alterations in fetal growth and birth weight is unquestionably important, given that both serve as predictive markers for the developmental health of a neonate and has been shown to be predictive of future health outcomes (Ward and Beachy 2003). Accurate measurement of PM_2.5_ exposure is difficult to obtain, and studies on this topic during pregnancy may be vulnerable to certain limitations. One limitation of research designs used in some of these studies on birth weight is the fact that results were drawn from absolute weight, not weight given gestational age (O’Donnell and Behie 2015). Some studies were also restricted to birth terms between 37–42 weeks (Holstius *et al* 2012). Additionally, most studies examined a single wildfire event and conducted analyses on this topic using air quality monitors or satellite images to assess environmental exposure (Holstius *et al* 2012), a method that may not be able to accurately account for microclimates, wind patterns and topographical differences within a given region. This study aims to more precisely measure maternal exposure to wildfire-specific PM_2.5_ throughout pregnancy at the ZIP code level in California to gain a more granular understanding of exposure to wildfire smoke and its effect on fetal growth. We hypothesize wildfire smoke exposure during pregnancy is associated with lower birth weight.

## 2. Methods

Our study population included births that occurred at the University of California San Francisco (UCSF) Betty Irene Women’s Hospital from January 1st, 2017 through March 12th, 2020. We extracted birth weight, gestational age, maternal age, maternal race/ethnicity, maternal education, prenatal care, parity, newborn sex, and Zone Improvement Plan ZIP code of maternal residence from UCSF’s Perinatal Database, which includes data from electronic health records and maintained for research purposes. We excluded pregnant people who did not receive prenatal care at UCSF (and were transferred likely due to complications, *N* = 987), smokers (*N* = 678), non-singleton pregnancies (*N* = 1641) and births that occurred after the WHO’s declaration of the COVID-19 pandemic in March 2020. We used birthweight and gestational age to calculate birthweight-for-gestational age *z*-scores using a US population (Talge *et al* 2014), which allowed us to examine birthweight adjusted for gestational age. We additionally considered categorical outcomes including LGA, defined as above the 90th percentile of birthweight-for-gestational age, and small for gestational age (SGA), defined as less than the 10th percentile of birthweight-for-gestational age.

Birth data were linked to daily wildfire attributable PM_2.5_ estimates for each ZIP code in the nine counties of the San Francisco Bay Area. Wildfire-specific PM_2.5_ was estimated using methods described previously in more detail (Aguilera *et al* 2023). In brief, we first estimated daily concentrations of total PM_2.5_ (from any source) at the ZIP code level using ensemble models that integrated multiple machine learning algorithms (e.g. random forest, gradient boosting and deep learning) and multiple predictor variables such as outdoor PM_2.5_ measurements from US EPA Air Quality System, aerosol optical depth, plume height, and meteorological variables (e.g. precipitation, temperature, wind speed and direction). We then used a spatiotemporal multiple imputation approach to estimate ZIP code-level wildfire PM_2.5_ concentrations (Aguilera *et al* 2023), which we aggregated to trimester average exposures. Non-wildfire PM_2.5_ concentrations were also estimated as the difference from the total measured PM_2.5_ concentrations.

We created both binary and continuous variables to capture different aspects of exposure, including an indicator for whether a pregnancy was exposed to any wildfire-specific PM_2.5_ and the average wildfire PM_2.5_ experienced over the duration of pregnancy and each trimester. We also recorded the number of days in each pregnancy that wildfire PM_2.5_ exceeded 5 *μ*g m^−3^ to capture exposure over a more intense wildfire smoke threshold.

We used linear regressions to estimate the association between each metric of wildfire smoke exposure and birthweight-for-gestational age *z*-scores and log-binomial regression for categorical outcomes including large- and SGA births. We considered potential confounders based on previous studies and subject matter knowledge (Abdo *et al* 2019). Thus, we adjusted our models with maternal age, level of maternal educational attainment, season of conception, and maternal race/ethnicity as a proxy for social vulnerability and potential experiences of discrimination.

We stratified by infant sex to examine potential effect modification, that is, differences in associations between wildfire PM_2.5_ and fetal growth among male and female infants. We used the Cochran Q test to determine if effect estimates were heterogeneous across infant sex strata.

## 3. Results

Our study population included 7923 births (table 1).

**Table 1.** San Francisco birthing study population demographics by exposure to wildfire-specific PM_2.5_ (wPM_2.5_) (*N* = 7923).

	Exposed to wPM_2.5_ (*N* = 4364)	Not exposed to wPM_2.5_ (*N* = 3559)	Total (*N* = 7923)
Maternal age (years)			
Mean (SD)	34.1 (4.62)	33.7 (4.70)	33.9 (4.66)
Median [Min, Max]	34.0 [16.0, 55.0]	34.0 [15.0, 51.0]	34.0 [15.0, 55.0]
Maternal race			
White	1999 (45.8%)	1603 (45.0%)	3602 (45.5%)
Black	219 (5.0%)	178 (5.0%)	397 (5.0%)
Latina	490 (11.2%)	419 (11.8%)	909 (11.5%)
Asian/Pacific Islander	1164 (26.7%)	939 (26.4%)	2103 (26.5%)
Other	492 (11.3%)	420 (11.8%)	912 (11.5%)
Maternal education			
No high school diploma	51 (1.2%)	36 (1.0%)	87 (1.1%)
Only high school	590 (13.5%)	531 (14.9%)	1121 (14.1%)
Some college	0 (0%)	0 (0%)	0 (0%)
Graduated college	1732 (39.7%)	1374 (38.6%)	3106 (39.2%)
Graduate school	1842 (42.2%)	1380 (38.8%)	3222 (40.7%)
Missing	149 (3.4%)	238 (6.7%)	387 (4.9%)
Newborn sex			
Male	2181 (50.0%)	1847 (51.9%)	4028 (50.8%)
Female	2183 (50.0%)	1712 (48.1%)	3895 (49.2%)
Parity			
1	2571 (58.9%)	2093 (58.8%)	4664 (58.9%)
2	1336 (30.6%)	1079 (30.3%)	2415 (30.5%)
3+	457 (10.4%)	387 (10.8%)	844 (10.6%)
Hypertension during pregnancy			
No hypertension	3714 (85.1%)	3002 (84.3%)	6716 (84.8%)
Hypertension	650 (14.9%)	557 (15.7%)	1207 (15.2%)
Type 2 diabetes during pregnancy			
No diabetes	4230 (96.9%)	3463 (97.3%)	7693 (97.1%)
Diabetes	134 (3.1%)	96 (2.7%)	230 (2.9%)
Health insurance type			
Private insurance	4218 (96.7%)	3433 (96.5%)	7651 (96.6%)
Medi-Cal/Medicaid	144 (3.3%)	125 (3.5%)	269 (3.4%)
Missing	2 (0.0%)	1 (0.0%)	3 (0.0%)
Birthsize for gestational age			
Large for gestational age	339 (7.8%)	299 (8.4%)	638 (8.1%)
Appropriate for gestational age	3656 (83.8%)	2940 (92.6%)	6596 (83.3%)
Small for gestational age	369 (8.5%)	320 (9%)	689 (8.7%)
Season of conception			
Fall	695 (15.9%)	561 (15.8%)	1256 (15.9%)
Spring	1290 (29.6%)	876 (24.6%)	2166 (27.3%)
Summer	1484 (34.0%)	1120 (31.5%)	2604 (32.9%)
Winter	895 (20.5%)	1002 (28.2%)	1897 (23.9%)

Our population was majority white (45.5%) and college-educated (79.9%). Average maternal age at birth was 33.9 years old. The non-white population was mostly Asian/Pacific Islander (26.5%), followed by Latinas (11.5%) and people whose racial groups were mixed race, Native American, or other (11.5%), and people who identified as Black (5.0%). Only a small percentage of our cohort were insured through Medi-Cal (3.4%), California’s form of Medicaid, compared to 42.1% of the birthing population in the U.S that used Medicaid to pay for labor and deliveries in 2019 (Martin *et al* 2022). A majority of people had no history of hypertension (84.8%) or diabetes at the time of their delivery (97.1%).

Out of the 7923 pregnancies in our cohort, over half (55.1%) were exposed to wildfire-specific PM_2.5_ in their residential ZIP code. Of the 4364 pregnancies that were exposed, the number of days of ambient wildfire PM_2.5_ averaged 6.86. During the study period (January 1st, 2017 and March 12th, 2020), peak wildfire-specific PM_2.5_ occurred during the Camp Fire in November 2018, where for 10 d straight PM_2.5_ levels exceeded 50 *μ*g m^−3^ in San Francisco and sometimes surpassed 100 *μ*g m^−3^.

We found a positive association between exposure to wildfire-specific PM_2.5_ smoke in the second trimester and increased birthweight-for-gestational age *z*-scores (table 2).

**Table 2.** Associations between wildfire-specific PM_2.5_ (wPM_2.5_) and birthweight-for-gestational age *Z*-score (*N* = 7923).

Wildfire-specific PM_2.5_ metrics	*ß* ^a^	95% CI
Average wPM_2.5_ over pregnancy	0.037	[−0.013, 0.087]
Average wPM_2.5_ in 1st trimester	0.002	[−0.026, 0.030]
^a^ Average wPM_2.5_ in 2nd trimester	0.039	[0.011, 0.067]
Average wPM_2.5_ in 3rd trimester	−0.009	[−0.037, 0.018]
Exposed to any wPM_2.5_	0.012	[−0.031, 0.054]
# of days wPM_2.5_ > 5 *μ*g m^−3^ in 1st trimester	0.001	[−0.008, 0.009]
^a^# of days wPM_2.5_ > 5 *μ*g m^−3^ in 2nd trimester	0.009	[0.001, 0.017]
# of days wPM_2.5_ > 5 *μ*g m^−3^ in 3rd trimester	−0.001	[−0.009, 0.007]
Total # of days wPM_2.5_ > 5 *μ*g m^−3^	0.003	[−0.001, 0.008]

^a^ Beta coefficient derived from linear regression for 1 *μ*g m^−3^ or 1 d of wildfire PM_2.5_ exposure adjusted for maternal age, education, race/ethnicity, season of conception.

This association was demonstrated both when exposure was measured as an average for PM_2.5_ in the second trimester (***ß*** = 0.039; 95% CI: 0.011, 0.067) as well as the number of days people experienced PM_2.5_ levels above 5 *μ*g m^−3^ (***ß*** = 0.009; 95% CI: 0.001, 0.017) in the second trimester. The Beta coefficient derived from linear regression for 1 *μg* m^−3^ or 1 d of wildfire PM_2.5_ exposure adjusted for maternal age, education, race/ethnicity, season of conception.

Average wildfire PM_2.5_ in the second trimester was associated with increased risk of LGA births (relative risk (*RR*) = 1.11; 95% CI: 1.02, 1.20), while average wildfire PM_2.5_ over the pregnancy duration was associated with decreased risk of SGA births (*RR* = 0.84; 95% CI: 0.60, 1.01). (table 3). The relative risk was derived from log-binomial regression for 1 *μ*g m^−3^ or 1 d of wildfire PM_2.5_ exposure and similarly was adjusted for maternal age, education, race/ethnicity, season of conception. Additionally, the total number of days people experienced PM_2.5_ levels greater than 5 *μ*g m^−3^ was associated with increased risk of LGA births (*RR* = 1.03; 95% CI: 1.00, 1.05) when exposed in the second trimester and a decreased risk of SGA births (*RR* = 0.98; 95% CI: 0.96, 1.01) when exposure was averaged across the entire pregnancy.

**Table 3.** Associations between wildfire-specific PM_2.5_ (wPM_2.5_) and large-for-gestational age (LGA) and small-for-gestational age (SGA) (*N* = 7923).

Large for gestational age		
Wildfire-specific PM_2.5_ metrics	*RR* ^a^	[95% CI]
Average wPM_2.5_ over pregnancy	1.13	[0.95, 1.33]
Average wPM_2.5_ in 1st trimester	0.96	[0.86, 1.06]
Average wPM_2.5_ in 2nd trimester	1.11	[1.02,1.20]
Average wPM_2.5_ in 3rd trimester	1.02	[0.92, 1.12]
Exposed to any wPM_2.5_	0.90	[0.78, 1.05]
# of days wPM_2.5_ > 5 *μ*g m^−3^ in 1st trimester	0.98	[0.95, 1.01]
# of days wPM_2.5_ > 5 *μ*g m^−3^ in 2nd trimester	1.03	[1.00,1.05]
# of days wPM_2.5_ > 5 *μ*g m^−3^ in 3rd trimester	1.01	[0.98, 1.04]
Total # of days wPM_2.5_ > 5 *μ*g m^−3^	1.01	[0.98, 1.03]
Small for gestational age		
Wildfire-specific PM_2.5_ metrics	*RR* ^a^	[95% CI]
Average wPM_2.5_ over pregnancy	0.84	[0.69, 1.01]
Average wPM_2.5_ in 1st trimester	0.95	[0.85, 1.05]
Average wPM_2.5_ in 2nd trimester	0.93	[0.83, 1.03]
Average wPM_2.5_ in 3rd trimester	0.97	[0.86, 1.07]
Exposed to any wPM_2.5_	0.93	[0.81, 1.08]
# of days wPM_2.5_ > 5 *μ*g m^−3^ in 1st trimester	0.99	[0.95, 1.02]
# of days wPM_2.5_ > 5 *μ*g m^−3^ in 2nd trimester	0.98	[0.96, 1.01]
# of days wPM_2.5_ > 5 *μ*g m^−3^ in 3rd trimester	0.99	[0.96, 1.02]
Total # of days wPM_2.5_ > 5 *μ*g m^−3^	0.98	[0.97, 1.00]

^a^ Relative risk derived from log-binomial regression for 1 *μ*g m^−3^ or 1 d of wildfire PM_2.5_ exposure adjusted for maternal age, education, race/ethnicity, season of conception.

Our sex-stratified analyses revealed a positive association between average wildfire-specific PM_2.5_ in the second trimester and increase in birthweight-for-gestational age *z*-score in females (***ß*** = 0.045; 95% CI: 0.005, 0.090); however, it was not statistically different from males (table 4).

**Table 4.** Infant sex-stratified associations between wildfire-specific PM_2.5_ and birthweight-for-gestational age *Z*-scores and *P*-value of Cochran *Q* test of homogeneity (*X* ^2^) (*N* = 7923).

	Female infants		Male infants		
Wildfire-specific PM_2.5_ metrics	*ß^a^*	[95% CI]	*ß^a^*	[95% CI]	*X^2^ p*-value
Average wPM_2.5_ over pregnancy	0.041	[−0.031,0.112]	0.034	[−0.035, 0.104]	0.90
Average wPM_2.5_ in 1st trimester	−0.009	[−0.050, 0.031]	0.014	[−0.025, 0.054]	0.41
Average wPM_2.5_ in 2nd trimester	0.045	[0.005, 0.090]	0.032	[−0.007, 0.071]	0.64
Average wPM_2.5_ in 3rd trimester	−0.001	[−0.041, 0.039]	−0.015	[−0.053, 0.022]	0.61
Exposed to any wPM_2.5_	−0.011	[−0.071, 0.052]	0.033	[−0.026, 0.092]	0.14
# of days wPM_2.5_ > 5 *μ*g m^−3^ in 1st trimester	−0.004	[−0.016, 0.009]	0.005	[−0.007, 0.017]	0.33
# of days wPM_2.5_ > 5 *μ*g m^−3^ in 2nd trimester	0.010	[−0.001, 0.022]	0.007	[−0.004, 0.018]	0.68
# of days wPM_2.5_ > 5 *μ*g m^−3^ in 3rd trimester	0.001	[−0.11, 0.011]	−0.001	[−0.012, 0.010]	0.72
Total # of days wPM_2.5_ > 5 *μ*m^−3^	0.003	[−0.004, 0.010]	0.004	[−0.003, 0.011]	0.85

^a^ Beta coefficient derived from linear regression for 1 *μ*g m^−3^ or 1 d of wildfire PM_2.5_ exposure adjusted for maternal age, education, race/ethnicity, season of conception.

According to the Cochran *Q* tests of homogeneity, male infants had stronger associations between increased birth weight and number of days with wPM_2.5_ above 5 *μ*g m^−3^ in the first trimester (*p* = 0.16) (table 4).

## 4. Discussion

Our results suggest a positive relationship between exposure to wildfire smoke and increased newborn birth weight, especially when exposure occurs in the second trimester. Analyses for births above the 90th percentile (LGA) and births below the 10th percentile (SGA) births were concordant with one another, indicating increased size following exposure in the second trimester. Notably, birth weight is correlated most strongly with fetal weight gain in the second trimester in singleton pregnancies (Rasmussen *et al* 2009), which may indicate that PM_2.5_ exposure in the second trimester corresponds to the window that fetuses are most susceptible to metabolic changes. Alternations in placental may play a role in this association with biological mechanisms including inflammation, oxidative stress, endocrine disruption, eoxyribonucleic acid (DNA) damage, telomere shortening, epigenetic changes, as well as metabolic, vascular, and endothelial dysregulation in the maternal-fetal unit (Basilio *et al* 2022).

While many studies have reported PM_2.5_ exposure in the second trimester with birth weight deviation, our results are contrary to most previous studies that have found an inverse relationship between wildfire smoke and neonatal birth weight. Our findings are consistent with one study from Australia demonstrating a positive association between PM_2.5_ exposure and increased birth weight among male infants; however, they did not specify timing of exposure (O’Donnell and Behie 2015). In our study, although the 95% confidence intervals of association included the null value, one exposure metric, number of days exposed to wPM_2.5_ above 5 *μ*g m^−3^ in the first trimester, was stronger among male infants according to the Cochran *Q* test of homogeneity. It is plausible that the positive associations between wPM_2.5_ and birthweight are stronger among male infants, but our study may have been underpowered to detect robust sex-specific associations. Evidence suggests that sex differences in fetal growth may be mediated by a difference in the regulation of gene expression and proteins between males and females in response to an adverse maternal environment (Clifton 2010) and in the context of maternal stress (Kaitz *et al* 2015), natural disasters (Bruckner *et al* 2010), and economic turbulence (Catalano *et al* 2005). Future studies should prioritize large sample sizes to maximize statistical power and assess sex differences following exposure to PM_2.5_.

There are significant methodological and demographic aspects of our study that may explain differences between our study and previous studies of birth weight associations in relation to exposure to wildfire and other types of air pollution. Previous studies on the effect of wildfire smoke and neonatal birth weight have used proximity to wildfire events or PM_2.5_ metrics from air monitors that do not distinguish between sources of PM_2.5_ emission, which can stem from the combustion of oil and diesel fuel in addition to wildfires (California Air Resources Board n.d). This exposure assessment was used to reduce exposure misclassification by removing other sources of PM_2.5_, such as car exhaust and industrial pollution. Additionally, it allowed us to obtain a more granular depiction of pregnant people’s PM_2.5_ exposure by using air quality measurements from the specific ZIP code in which they lived during their pregnancy, which provided us more location-specific data than PM_2.5_ levels from air pollutant monitors in each region. Nonetheless, similar to previous studies, we lack information on time activity, indoor infiltration, and mobility, which may affect exposure misclassification. Future prospective studies may be equipped to ask such questions and incorporate these data. Furthermore, although few studies have examined short term exposures with respect to birth outcomes, shorter averaging periods may be better suited to examine potential critical periods of exposure, and minimize exposure misclassification (Tanner *et al* 2016).

Our findings may be limited by the fact that UCSF serves a disproportionately privileged population, who may be afforded the option to leave the area or stay indoors with adequate filtration when air quality is poor. While our patient population was largely white (45.5%) and college educated (79.9%), people of color comprise a disproportionate amount of the working class population (Rowell 2018), which is a group that is more likely to be denied work-based health insurance, sick leave, and maternity or paternity leave (Desilver 2020). Given the lack of universal health care in the United States, there exists a significant disparity in access to affordable medical services and workplace flexibility in the event of pregnancy, chronic health conditions, or natural disasters like wildfires. The Pew Research Center estimates that less than a third of workers within the lowest tenth of earned income receive benefits often afforded to the top tier workers (Desilver 2020), which may disincentivize workers from missing work, even when conditions are hazardous for their health. Employers have repeatedly neglected to protect workers from toxic ambient smoke exposure during wildfire season in California (Romero 2021), even when nearly 4 million Californians work in industries—like agriculture, construction and utilities (California Employment Development Department, n.d)—that require them to be outside. Furthermore, there are indications throughout the environmental health literature that being white and upper-class are protective factors, and that poor communities of color, as well as people who work in lower-income occupations, are more at risk (Shrestha *et al* 2019). Our study population may not be representative of Californians’ typical wildfire exposure risks, though exposures are expected to increase owing to climate change. However, our study is not generalizable and may have been able to avoid exposure with their resources. Future studies should address the socioeconomic and racial disparities of wildfire smoke exposure, as such analyses are imperative for understanding the vulnerabilities of different communities to poor birth outcomes. Additionally, they can aim to address the role that structural racism, housing inequality, and socioeconomic vulnerability play in determining how much a pregnant person is exposed to wildfire smoke, and how this may contribute to disparities in birth outcomes (Sklar and Padula 2022).

## 5. Conclusion

Our study added to the small, but growing literature on the impact of wildfire-specific PM_2.5_ exposures during pregnancy and birth weight. More studies are needed in diverse populations to elucidate the associations for fetal growth and additional adverse birth outcomes. In the meantime, we need to advocate for policies that ensure workplace accommodations such as paid sick leave and pregnancy/maternity leave, and accommodations during wildfire (or other climate-related disasters) must be implemented to protect the most vulnerable portions of the pregnant population from experiencing adverse birth outcomes related to wildfire smoke exposure.

## Data Availability

The data cannot be made publicly available upon publication because they contain sensitive personal information. The data that support the findings of this study are available upon reasonable request from the authors.
